# Protocol of a Pilot Study of Technology-Enabled Coproduction in Pediatric Chronic Illness Care

**DOI:** 10.2196/resprot.7074

**Published:** 2017-04-28

**Authors:** Heather C Kaplan, Sunny Narendra Thakkar, Lisa Burns, Barbara Chini, Dana MH Dykes, Gary L McPhail, Erin Moore, Shehzad Ahmed Saeed, Ian Eslick, Peter A Margolis, Lisa Opipari-Arrigan

**Affiliations:** ^1^ Cincinnati Children's Hospital Medical Center James M. Anderson Center for Health Systems Excellence Cincinnati, OH United States; ^2^ Cincinnati Children's Hospital Medical Center Perinatal Institute Cincinnati, OH United States; ^3^ Cincinnati Children's Hospital Medical Center Division of Pulmonary Medicine Cincinnati, OH United States; ^4^ Cincinnati Children's Hospital Medical Center Division of Gastroenterology, Hepatology, & Nutrition Cincinnati, OH United States; ^5^ Dayton Children’s Hospital Department of Gastroenterology and Nutrition Dayton, OH United States; ^6^ Vital Labs, Inc. San Francisco, CA United States; ^7^ Cincinnati Children's Hospital Medical Center Behavioral Medicine & Clinical Psychology Cincinnati, OH United States

**Keywords:** mobile applications, chronic disease, physician-patient relationship, cystic fibrosis, inflammatory bowel disease

## Abstract

**Background:**

Pediatric chronic illness care models are traditionally organized around acute episodes of care and may not meet the needs of patients and their families. Interventions that extend the patient-clinician interaction beyond the health care visit, allow for asynchronous and bidirectional feedback loops that span visits and daily life, and facilitate seamless sharing of information are needed to support a care delivery system that is more collaborative, continuous, and data-driven. Orchestra is a mobile health technology platform and intervention designed to transform the management of chronic diseases by optimizing patient-clinician coproduction of care.

**Objective:**

The aim of this study is to assess the feasibility, acceptability, and preliminary impact of the Orchestra technology and intervention in the context of pediatric chronic illness care.

**Methods:**

This study will be conducted in the cystic fibrosis and inflammatory bowel disease clinics at Cincinnati Children’s Hospital Medical Center. We will enroll interested patients and their caregivers to work with clinicians to use the Orchestra technology platform and care model over a 6-month period. In parallel, we will use quality improvement methods to improve processes for integrating Orchestra into clinic workflows and patient/family lifestyles. We will use surveys, interviews, technology use data, and measures of clinical outcomes to assess the feasibility, acceptability, and preliminary impact of Orchestra. Outcome measures will include assessments of: (1) enrollment and dropout rates; (2) duration of engagement/sustained use; (3) symptom and patient-reported outcome tracker completion rates; (4) perceived impact on treatment plan, communication with the clinical team, visit preparation, and overall care; (5) changes in disease self-efficacy and engagement in care; and (6) clinical outcomes and health care utilization.

**Results:**

Participant recruitment began in mid-2015, with results expected in 2017.

**Conclusions:**

Chronic disease management needs a dramatic transformation to support more collaborative, effective, and patient-centered care. This study is unique in that it is testing not only the impact of technology, but also the necessary processes that facilitate patient and clinician collaboration. This pilot study is designed to examine how technology-enabled coproduction can be implemented in real-life clinical contexts. Once the Orchestra technology and intervention are optimized to ensure feasibility and acceptability, future studies can test the effectiveness of this approach to improve patient outcomes and health care value.

## Introduction

Effective management of chronic illness requires a different type of health care delivery system than presently exists [1]. Our current models of care, which are organized around treating acute episodic conditions, do not meet the needs of the growing number of patients with serious, chronic health problems [2,3]. Typically, individuals with chronic illness are seen by their specialist a few times a year. During these brief visits, patients and families are expected to identify, remember, and communicate to the clinical team the most relevant aspects of their illness experience from the previous weeks or months. Clinicians are then charged with gathering enough detail from the information shared by patients and families to make optimal treatment recommendations. Traditional clinic visits provide the clinician with only a blurry and fleeting snapshot of a patient’s disease [4]. The experience is similar for patients and families, who generally lack access to clinical data and test results and, even when results are made available, they are not accompanied by understandable explanations. These issues hamper patients’ ability to fully participate in decision-making [4].

While the clinical encounter is a valuable interaction, its design does not support optimal patient engagement, clinician efficiency and effectiveness, or health outcomes [1,3-5]. To improve the clinical encounter, we need ways to: (1) easily extend patient-clinician interaction beyond the circumscribed visit; (2) allow for asynchronous, bidirectional feedback loops that span both visits and daily life; (3) regularly collect and monitor patient-reported data between visits; and (4) seamlessly share clinically-generated and patient-generated information [2,4]. In addition, patients and clinicians need support to truly coproduce care, allowing them to collaborate to produce *information* (eg, clinical data, patient-reported outcomes [PROs]), *knowledge* (insights), and *know-how* (expertise) to improve health care and health outcomes [1,3-5].

Technology can help to address these gaps by enabling low-friction data collection, sharing, and communication [3,6-8]. An increasing number of technologies designed to support chronic disease management are being developed. A recent study of an electronic health record (EHR)-linked patient portal to track family treatment concerns, goals, symptoms, medication side-effects, and adherence in pediatric asthma showed promise for improving communication, self-management, and outcomes [9]. In addition, there is an exponential rise in reports of apps being developed to support better chronic disease management in diabetes [10-12], hypertension [13], chronic pain [14]. However, technology is only part of the solution. A recent systematic review that examined the effectiveness of mobile health (mHealth) in supporting chronic disease management found mixed results [15]. The authors concluded that while the potential for improved outcomes is high, more work is needed to focus on how technology is implemented in ways that overcome existing barriers [15]. Technology alone will have minimal impact on chronic disease management unless it is seamlessly integrated into patients’ lives and clinicians’ workflows in a way that relieves burdens and addresses unmet needs [6,7].

Orchestra is an mHealth technology platform (mobile, tablet, desktop) and intervention designed with and for patients, families, and clinicians to facilitate coproduction of care. Orchestra is aimed at transforming the management of chronic disease by making care more collaborative, continuous, and effective. Orchestra emerged from goal-directed design work conducted as part of the development of a collaborative chronic care network for pediatric inflammatory bowel disease (IBD) [16]. Interviews and direct observations led to the creation of personas and scenarios that were used to derive requirements of an improved chronic care system. The personas and scenarios guided the generation of 100 prototype interventions designed to address these requirements. Among these prototypes were: (1) the Personalized Learning System [17], a Web-based and short messaging system (SMS) technology platform that enabled patients with chronic diseases to work collaboratively with their clinicians to identify issues of importance to them, track outcomes, and learn from both routine changes in everyday life (eg, diet changes, sleep patterns) and formal planned experiments; and (2) the E^3^(Engaged, Empowered, Electronic) Healthcare Study [18], which developed and tested mobile and Web-based tools, including a previsit planner and weekly symptom tracker designed to optimize clinical interactions and shared decision-making. Observations and learnings from these two prototypes were used to inform the design and development of the Orchestra technology platform and intervention.

The aim of this pilot study is to assess the feasibility, acceptability, and preliminary impact of Orchestra in the context of pediatric chronic illness, while simultaneously developing and refining the processes that allow for integration into the care delivery system [19].

## Methods

### Study Setting

This study is being conducted in the Cystic Fibrosis (CF) Center and IBD clinic at Cincinnati Children’s Hospital Medical Center (CCHMC). CCHMC is a 629-bed children’s hospital with associated ambulatory clinics, and is the only children’s hospital in the Cincinnati metropolitan area (population 2.3 million). The CF clinic within the pulmonology division cares for approximately 225 patients with CF and is one of 10 CF Foundation Research Centers in the United States. The Schubert Martin IBD Center cares for approximately 700 patients with Crohn’s disease and ulcerative colitis, and is home to a wide array of cutting-edge basic and translational research. Both clinics are staffed with multidisciplinary teams that include psychology, nutrition, and social work. CCHMC uses Epic (Verona, Wisconsin) as its EHR.

### Project Timeline

The development of the Orchestra technology and intervention has proceeded through a series of small pilot studies, using a rapid iteration process. The first phase of pilot testing was designed to refine the technology (June to December 2014). The platform was tested with 4 physicians, 17 patients, and 31 caregivers. The current phase of testing (phase 2) is designed to: increase the number and type of clinicians involved (physicians, nurses, dietitians, social workers, psychologist, and respiratory therapists); increase the number of patients using Orchestra; refine the processes necessary to integrate Orchestra into patients’ lives and clinicians’ workflows; and measure the feasibility, acceptability, and impact of the Orchestra intervention when used over a 6-month period. Phase 2 aims to enroll 100 participants.

### Study Intervention

#### Overview

The Orchestra technology and intervention is designed to: (1) extend clinical data sharing and decision-making beyond the circumscribed clinic visit by allowing ongoing, asynchronous, and bidirectional symptom monitoring and feedback loops between clinicians and patients; (2) support patients and clinicians in preparing for the clinic visit and having the right information at the right time so that they can effectively collaborate, communicate, and codesign a care plan that works; (3) prepare patients and clinicians to be equal partners in a culture of medicine in which they have not been trained to do this; (4) transform the valuable time spent during the clinic visit from information transfer based on ambiguous memory and limited recall to data-based problem-solving that addresses patient and family goals; and (5) support patients and clinicians in using data, quality improvement methods, and planned experimentation to learn about which treatments and lifestyle modifications help them feel better. Screenshots of the Orchestra technology components are provided in Multimedia Appendix 1.

#### Technology Components

##### Symptom Tracking and Journal/Note Entry

This feature allows patients/caregivers to regularly track symptoms, health behaviors, and standardized multi-item PRO measures between clinic visits. Frequency of data tracking can range from daily to weekly, based on the specific measure and the schedule agreed upon by the patient/caregiver and clinician. Tracking is done on the mobile app or the desktop platform and can be prompted through push notification, SMS, and/or email. Tracked symptoms are depicted via line graphs and are viewable in real-time by patient/caregiver and clinician. Patient/caregiver and clinician can attach a note to a specific data point and can capture general thoughts in journal entries. Notes and journals are designed to document observations about the data that facilitate learning from variation and natural experiments (eg, impact of travel or a diet change) and enhance communication. Symptom tracking is meant to improve clarity regarding the patient’s condition and refine hypotheses about changes that might improve the patient’s symptoms. Examples of participants’ symptom trackers are provided in Multimedia Appendix 2.

##### Automated Signals for Changes in Patient Status

Clinicians are notified of changes in a patient’s symptoms when certain preestablished criteria are met. This feature is meant to alert clinicians to data that is potentially actionable. Clinicians can enable and customize automated signals for each individual measure being tracked by the participant. Data signals are sent to clinicians via email and generated using two distinct methods. The statistical process control (SPC) option generates signals by using statistical algorithms to identify significant changes in the individual measures. Once a sufficient amount of baseline data has been entered by the participant (usually the first 20 data entries) [20], it is continuously analyzed using SPC rules to identify significant changes. Alternatively, the threshold option employs user-defined formulas to signal clinicians if established criteria are met (eg, if frequency of coughing is greater than *most of the day* for more than three days in a row). By default, measures are set up with signals turned off. Clinicians have the option of enabling one or both types of signals on measures that are key symptom indicators of a patient’s health status. At this time, patients and caregivers are not notified of signals.

##### The Previsit Plan

A previsit plan (PVP) is pushed to the patient’s/caregiver’s account two weeks prior to a clinic appointment, and consists of two components: (1) a health metric review with personalized feedback, and (2) a previsit survey. In the health metric review, laboratory and other relevant health metrics from the previous 12 months (obtained from the HER) are displayed using intuitive visualizations and plain language to help patients/caregivers understand the meaning of their results and health trends. Results are accompanied by personalized suggestions of topics to address during the clinical encounter, based on the results. This component contains health metrics specific to the relevant chronic condition. For example, in IBD key metrics include variables such as hematocrit, serum albumin, height, weight, body mass index (BMI), and physician global assessment of disease activity. CF key metrics include the percent predicted Forced Expiratory Volume in 1 second (FEV1) and BMI percentile. The previsit survey graphically depicts all responses to tracked data during the intervisit period, prompts the patient/caregiver to respond to a set of questions related to current symptoms and functioning, provides the patient/caregiver with an opportunity to review all notes and journal entries recorded during the intervisit period and select the ones that the participant would like to discuss with their clinicians, and solicits goals for the upcoming visit. The previsit survey, once complete, is viewable in real time by the patient/caregiver and by the clinician. The clinician view includes flagging of responses outside of normal limits.

##### Planned Experimentation

Patients/caregivers and clinicians are supported in using quasi-experimental or experimental designs to learn from data tracking and test hypotheses. Experimental designs can range from simple pre/post designs to more formal N-of-1 studies using multiple cross-over designs. Participants and clinicians work together to test hypotheses related to starting new medications or making diet and lifestyle changes.

##### Clinician Portal

Clinicians view their Orchestra patients via a Web-based portal. Clinicians can access the portal, using one-click single-sign-on authentication through the EHR, to support seamless workflow integration. A basic population panel includes patient name, age, gender, names of individuals tracking (patient and/or caregiver), and date of the next scheduled visit (pulled from the EHR). The population panel enables simple hierarchical sorting of patients by clinician, care team, and clinic, and serves as the gateway to accessing detailed symptom-tracking graphs, journals/notes, and PVPs.

#### Intervention Components

##### Shared Decision-Making Regarding Goal for Use

As a participant is introduced to Orchestra, the clinician guides a collaborative discussion with the patient/caregiver about identifying a clear purpose for how Orchestra will be used to benefit the patient’s care, and which are the best measures to track, in order to achieve the shared goal.

##### Establishment of a Social Contract

As a participant is introduced to Orchestra, the clinician guides a discussion on how often the clinical team will look at the data. The *social contract* supports expectation-setting around frequency of communication, helps participants understand that the clinical team values the data and is looking at data during the intervisit period, and supports clinicians in using the platform during the intervisit period *.*

##### Reinforcing Use During Intervisit Period

Clinicians review all data signals during the intervisit period and reach out to the patient/caregiver as appropriate. Clinicians are encouraged to review and refer to Orchestra data prior to or during any intervisit phone or email communication with a patient/caregiver. Clinicians are encouraged to incorporate the PVP into their preclinic planning process in order to be apprised of the patient’s current status, and be prepared to address patient goals at the visit. In addition, clinicians are encouraged to use the Orchestra journaling feature to leave notes of encouragement related to system use for participants.

##### Collaborative Review of Tracked Data and Previsit Plan at Clinic Visits

Clinicians are encouraged to refer to Orchestra tracker data and the PVP during the clinical encounter to make the patient/caregiver aware that they have reviewed the data and that it is contributing to clinical decision making, and to reinforce system use. Clinicians are also encouraged to jointly review the Orchestra graphs with patients/caregivers during the encounter as a tool for education and collaboration.

### Study Population

Participants are eligible for the study if they (1) are able to speak/read English, (2) have a clinical diagnosis of CF or IBD, (3) are between the ages of 14 and 21 years and/or are parents or legal guardians of patients between the ages of birth and 21 years, (4) have a smartphone or tablet device with compatible iOS or Android operating system, (5) have a mobile data plan and/or Internet connection, (6) have at least two clinic visits during the period of tool deployment (one at study entry, and at least one subsequent visit within 6 months), and (7) do not have a comorbid disorder that prohibits participation.

### Ethics

The protocol, all research staff, and all patient-facing materials related to the study were approved by the Institutional Review Board (IRB) at CCHMC. Data security was addressed as part of the IRB review and is managed in three ways. First, the Orchestra platform is hosted on a cluster of dedicated machines isolated within a virtual private cloud (VPC). Second, data are fully encrypted in transit between machines within the VPC. Simple messages that do not carry protected health information (PHI) are sent by the system, and unidentifiable numeric responses are typically returned. The only PHI involved in the transaction is the phone number or unique identification (ID) used to identify the participant’s phone. Participants are able to opt out of these mechanisms and respond entirely through a secure website if they choose. Third, the Orchestra platform makes selective, secure connections to CCHMC Web services for the specific purposes of gathering laboratory values and visit-scheduling information, and registering new participants. Registration activities pass a short set of personal identifiers (eg, name, date of birth, and cell phone number) to the platform and the platform returns a unique ID to Epic, which is used to open the platform within Epic and access laboratory data.

### Study Procedures

**Figure 1:**
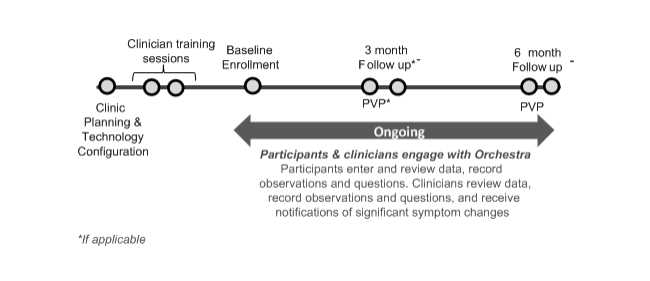
Study Procedures.

This is a prospective, single group, pre/post pilot study. Study procedures are summarized in Figure 1 and are broken down into the following phases: *Clinic Planning*, *Clinician Onboarding and Training*, *Participant In-Clinic Training and Onboarding (Baseline Visit)*, *Intervisit Period*, *Follow-Up Visits*, *Ongoing Process Improvements for Integrating Orchestra in the Clinic*, and *Technology Support.*

#### Clinic Planning

Select clinicians in the CF and IBD clinics (ie, clinician champions) partnered with the research team to develop a standard, condition-specific catalogue of relevant measures for participants to track. Validated measures were used when available; otherwise new questions were written to assess relevant symptoms and behaviors. Measures were preloaded into the Orchestra platform for ease of tracker selection and set up. Clinical champions selected the previsit survey questions and health metrics for the PVP, developed text for clinical algorithms that describe what each health metric means, and suggested topics based on the patient’s results to discuss at the visit. The planning phase also included the development of process maps defining how Orchestra integrates into clinic visits and intervisit workflows, including when and how potential participants are introduced to the study, how clinicians work with their patients/families to select trackers, when and how patients are set up and oriented to the Orchestra technology, and how Orchestra data are integrated into the care process to facilitate coproduction.

#### Clinician Onboarding and Training

All clinicians that cared for patients, including physicians, nurses, dietitians, respiratory therapists, and social workers, received training on the use of Orchestra. Two 90-minute, condition-specific, in-person training sessions were conducted. Training included instructions on how to use the Orchestra technology platform and how to incorporate the Orchestra intervention into patient care. Clinicians received individual accounts with clinic-level access to the Orchestra platform that allowed them to access all patients using Orchestra in their clinic.

#### Participant In-Clinic Training and Onboarding (Baseline Visit)

Three main methods of study recruitment are employed, and the recruitment method varies depending on the clinic flow and scheduling. Eligible participants are either identified and contacted by study and/or clinic staff prior to an upcoming appointment or, if not contacted in advance, approached in-person at the clinic appointment. Recruitment is completed with interested participants at the clinic visit. Research staff meets with all interested caregivers and patients to review and complete informed consent. Assent is obtained from all children aged 14-17 who are participating in the study. Consent is obtained from parents (or legal guardians) or study participants who are 18 years of age or older.

After clinic check-in, eligible participants are shown a 2-minute introductory video designed to spark interest in using Orchestra. This video focuses on inspiring patients to visualize a redesigned approach to care. Research coordinators (RCs) initiate study enrollment and inform the clinical team of a patient’s/caregiver’s interest in using Orchestra. During the clinical encounter, participants collaborate with their clinicians to identify goals for Orchestra use and select trackers and tracker frequency (eg, daily, weekly, other). Upon completion of the visit, clinicians independently decide whether to set data signals, and the type of signal to set (SPC and/or threshold), on any of the selected trackers in order to be alerted to a change in patient status.

After the clinical encounter, the RC meets with the participant to complete baseline surveys. The RC creates an Orchestra participant account via administrator functionality on the platform and supports the participant in downloading and setting up the app on their mobile device before they leave the clinic. After installation, the RC ensures that the account is functioning correctly and the participant is comfortable with basic app use. The app was developed with strong user interface design principles, and we anticipate that minimal training will be necessary to ensure patients can use the Orchestra app features. All participants receive a verbal review of the Orchestra features (during the consent process) and a paper-based quick start guide (included in Multimedia Appendix 3). In-depth tutorials are provided by RC coordinators if requested by a patient or family, or if the RC feels the patient needs additional instruction.

#### Intervisit Period

At the specified time and frequency, participants receive notifications to answer their selected trackers via their mobile device. Participants’ responses to trackers are displayed graphically, in real-time, on the app and on the Web-based platform. Line graphs display all data points chronologically with zoom in/out functionality. On the clinician-facing Web interface only, graphs include a median or mean line describing the central tendency of the data. Continuous measures also display control limits reflecting approximately 3 standard deviations from the mean, based on SPC rules. During the intervisit period, participants and clinicians are encouraged to record observations or questions by annotating data points, or via journal entries.

Clinicians are notified of significant changes in symptoms by email if SPC and/or threshold signals are set and the symptom data meet specified firing criteria. At any time during the intervisit period, if a participant shows a pattern of nonresponse (eg, is not completing daily trackers for 3 days in a row or weekly trackers for 2 weeks in a row), an RC contacts the participant to determine if the participant is experiencing technical problems or any other barriers to Orchestra use. As established in the social contract during the initial participant setup, check-ins are completed by the participant and clinician at the predetermined frequency. Check-ins are completed via MyChart (a patient communication portal available via the Epic EHR), email, phone, in-person, or using the journaling feature of the Orchestra app.

The PVP is automatically pushed to a participant’s account approximately two weeks prior to a scheduled clinic visit. Participants are notified via their mobile device to review and complete the PVP. Clinicians are notified via email when a PVP is completed, and completed PVPs can be accessed at any time via the Orchestra platform.

#### Follow-Up Visits

Study follow-ups occur during routine clinic visits at 3 months (if applicable) and 6 months after enrollment. All participants are expected to have at least one follow up visit within 6 months of enrollment, based on standard clinical follow-up recommendations for IBD and CF. During the follow-up visit, clinicians and participants are encouraged to collaboratively review the PVP, symptom trackers, and journal entries and notes. Participants and clinicians are also encouraged to identify any changes in Orchestra setup (ie, tracker frequency, addition of a new tracker, discontinuation of a current tracker) that are needed to better learn from the data. After the clinical encounter, the RC meets with the participant to complete follow-up surveys and a structured qualitative interview.

#### Ongoing Process Improvements for Integrating Orchestra in the Clinic

Weekly standing meetings involving the research team and clinician champions from the CF and IBD clinics are held to discuss approaches to improve clinical processes, including: (1) how the patient/caregiver is introduced to Orchestra; (2) how clinicians are supported in using shared decision-making principles when discussing Orchestra during the clinical encounter; (3) patient technology setup (ie, Orchestra app install); and (4) workflows for managing the intervisit period. We use quality improvement methods to improve processes.

#### Technology Support

RCs are the first-line responders to technology issues reported by participants and clinicians. RCs attempt to solve barriers to using the technology and, if unsuccessful, report the technology issue to Vital Labs, Inc., which attempts to resolve the issue within 48 hours. RCs track the number and type of technology issues (*bugs*) to inform future improvements of the technology and develop more scalable processes for technology support.

### Data Collection

#### Data Sources

Data are collected from participants via several sources, including: *Participant Assessments (Survey/Interview)*, *Clinician Assessments (Survey)*, *Orchestra Data*, and *Electronic Health Record/Clinical Outcome Data.*

##### Participant Assessments (Survey/Interview)

Enrolled participants complete surveys either at a scheduled clinic visit or via mail/email immediately following a clinic visit. Participants complete up to three assessments: baseline, 3-month (if applicable), and 6-month follow-up visits. Surveys are completed electronically via iPads or laptops directly linked to a Research Electronic Data Capture (REDCap) database for instant and secure electronic data storage. In-person interviews are conducted by the RC at the follow-up visits using a structured interview guide. Questions are designed to assess participants’ experience with the tools, including how they used Orchestra, barriers and facilitators of use, ways in which Orchestra improves the patient’s health status and experience with care delivery, and how the tool and intervention could be improved. RCs audio-record interviews and take written notes immediately following the interviews.

##### Clinician Assessments (Survey)

At each study visit, the patient’s physician and, if applicable, a second clinical team member involved in using Orchestra with the patient complete surveys. Clinician surveys are emailed to each clinician via the REDCap survey administration feature.

##### Orchestra Data

Using the Orchestra mobile app and Web-based platform, participants input data, including but not limited to, self-report of symptoms and behaviors and validated PRO measures. Participants and clinicians can also enter journal comments and notes using the Orchestra platform. All data entered into the Orchestra platform are available to export in standard file formats.

##### Electronic Health Record/Clinical Outcome Data

Basic demographic information and data on patient outcomes are collected from the patient medical record and entered into the REDCap database. In addition, for the subset of patients who have a data alert signal during the study period, RCs review the EHR to determine if the data alert signal leads to action or a change in treatment plan. If unable to determine whether action occurred or if the action is related to the signal, the RC contacts the clinician to verify.

#### Measures

Demographic data are collected from all participants 18 years or older and caregivers provide data for patients under 18. We record the participants’ age, race, ethnicity, gender, education, socioeconomic status, and familiarity with (and usage of) various technology platforms (eg, blogging, texting, using a tablet, video chatting). We also collect data on the patients’ age at diagnosis and any comorbid medical conditions from the EHR.

We are evaluating fidelity to the Orchestra intervention by determining the extent to which the key components of the intervention are used, including selection of goal and measures, completion of PVP, in-clinic discussion of tracked data/PVP, and follow-up of signals generated by the Orchestra system. We are also examining how participants choose to configure Orchestra to meet individual patient and clinician goals by measuring: (1) who has elected to track (adolescent patients vs caregivers vs both), (2) how many symptoms they have chosen to track, (3) the most common symptoms tracked, and (4) use of the surveillance versus planned experimentation features. The main quantitative outcome measures (Table 1) are designed to assess *Feasibility*, *Acceptability*, and *Clinical Impact.*

**Table 1:** Quantitative outcome measures.

Measure		Baseline	3-month Visit	6-month Visit	Study Duration
**Feasibility**					
	Set up time for app^a^	X			
	Added burden to clinical visit (subjective)	X			
**Acceptability**					
	Enrollment rate^b^	X			
	Drop-out rate^b^				X
	Duration of engagement^b^				X
	Orchestra use (eg, chart views, use of notes, journals)				X
	Tracker completion rate				X
	Completion rate of PVP survey		X	X	
**Clinical Impact**					
	Perceived value		X	X	
	Improved treatment plan		X	X	
	Improved communication		X	X	
	Positive impact on care		X	X	
	Improved clinic preparation		X	X	
	Utility of the tools		X	X	
	Improved disease insight		X	X	
	Percent of signals resulting in change in care plan^c^				X
	Disease self-efficacy (change from baseline)	X	X	X	
	Participant engagement (change from baseline)	X	X	X	
	Number of hospitalizations				X
	Number of emergency department visits				X
	Weight, BMI, BMI percentile *(CF)*	X	X	X	
	FEV1 *(CF)*	X	X	X	
	Number of pulmonary exacerbations *(CF)*				X
	Number of intravenous antibiotic courses *(CF)*				X
	Days in remission *(IBD)*				X
	Days in sustained remission *(IBD)*				X

^a^Includes app download/installation, account and tracker configuration, delivery of the quick start guide, and verbal instruction for select patients.

^b^Measure of both feasibility and acceptability.

^c^Based on review of the EHR and/or discussion with clinician.

##### Feasibility

We are evaluating the feasibility of the Orchestra intervention and technology platform in a clinical environment. We are assessing the costs and benefits of adding Orchestra into the clinical workflow, including added time during the encounter and impact on visit and intervisit care management.

##### Acceptability

The acceptability of the Orchestra intervention and technology is being determined by examining the participants’ tracker completion rate and the completion rate of the PVP. Other measures of acceptability include dropout rate (including participants who withdraw or those that are lost-to-follow-up) and sustained use over time.

##### Clinical Impact

We are evaluating the preliminary impact of the Orchestra intervention on outcomes, as reported by patients/caregivers and clinicians. We are assessing the perceived utility of Orchestra and impressions of the impact of Orchestra on care, collaboration, preparation and involvement in the clinic visit, disease insight, and treatment plan quality. We are also assessing changes in participant engagement in the visit and disease self-efficacy [21] from baseline to follow-up visits. Impact on the treatment plan is being measured objectively by examining whether data signals lead to action or change in treatment plan. We are also examining impact on clinical outcomes and health care utilization, although we anticipate that changes in these outcomes may not be realized in a short 6-month intervention.

Self-efficacy is being measured with the validated Self Efficacy for Managing Chronic Disease Questionnaire [21]. Engagement in the clinic and impact of using the Orchestra tool on care quality, patient-clinician collaboration, visit preparation, disease insight, treatment plan, and the tool’s perceived usefulness are being assessed using a novel survey developed specifically for this study. Survey questions are answered using a 6-point scale ranging from *Strongly Disagree* (1) to *Strongly Agree* (6).

#### Sample Size Calculation

The goal sample size of 100 was determined based on knowledge of the eligible population in each clinic, frequency of clinic visits, available research resources, and a desire to complete the study within the span of one year while the technology remains current. As efficacy is not the primary endpoint, we did not perform an *a priori* power calculation.

#### Statistical Analyses

##### Quantitative Data

Version 24 of the IBM SPSS Statistics program will be used to perform all data analyses. Participant characteristics will be summarized and described. Measures of fidelity, feasibility, acceptability, and proximal clinical impact of the Orchestra intervention and technology platform that are obtained only at follow up-visits (eg, after the start of the intervention) will be summarized with descriptive statistics, including means and ranges for continuous variables and percentages for categorical variables. Data will be described separately for 3-month and 6-month follow-up visits because some patients may have only a 3-month or 6-month follow up-visit. For measures that are being assessed at both baseline and follow-up (eg, measures of disease self-efficacy and visit engagement), we will use paired t-tests to compare outcomes at the beginning and end of participation. We will use *t*-tests and the Cohen *d* statistic to determine the impact of condition (CF vs IBD), person tracking (caregiver or child), and baseline health status. Univariate analyses of variances will be used to assess participants’ tool usage across study clinicians and across baseline health status. Pearson Correlation Coefficients will be used to examine relationships between tool use and perceived impact on care. Data regarding changes in health outcomes will be hypothesis-generating, as we do not expect impact on disease outcomes in a 6-month time frame.

##### Qualitative Data

The research team will review and code notes and audio recordings from patient interviews in addition to the log of issues, problems, and suggestions maintained by the research team. We will identify themes that emerge from the data to develop a better understanding of the experience of engaging with the Orchestra technology platform and intervention, and a more complete view of the ways in which this complex intervention impacts the patient’s health status and experience with care delivery. Qualitative data will be used to enrich the quantitative data collection by providing a deeper and better understanding of acceptability, feasibility, and impact.

## Results

Participant recruitment for phase 2 pilot testing began in May 2015 and continued until May 2016. Follow-up data collection was completed in autumn 2016. Results are expected in 2017.

## Discussion

The clinical encounter is the setting in which outpatient health care happens; however, we are far from maximizing its potential to improve patient understanding, clinician efficiency and effectiveness, patient-clinician collaboration, and health outcomes [3,4]. mHealth technology has the potential to support a care delivery system that enables patients and clinicians to work together to create more collaborative, continuous, proactive, data-driven, and effective care [3,6-8]. However, despite the growth in mHealth technology, we have yet to see transformation in the way care is delivered to individuals with chronic illnesses [22]. By its very nature, chronic care delivery is always shared work (eg, coproduced) between patient and clinician [5], yet the vast majority of mHealth technology is built to facilitate the work of either patients (ie, personal health record, health tracking) or clinicians (ie, communication among colleagues, access to drug information, continuing education), as opposed to enabling patients and clinicians to collaboratively work together to coproduce better health and health care [6,7].

This study is designed to test the feasibility, acceptability, and preliminary impact of an mHealth technology platform and intervention aimed at transforming the management of chronic diseases and facilitating coproduction between patients and clinicians. Orchestra is designed to make care more collaborative, continuous, and effective by enabling symptom/wellness tracking with real-time data visualization and sharing between patients and clinicians, automated symptom surveillance with actionable alerts to signal potential changes in patient status, collaborative previsit planning that includes personalized lab results and health metric feedback for patients, and opportunities for planned experimentation. While Orchestra can enable low-friction data collection, sharing, and communication, it will have minimal impact on chronic disease management unless it integrates seamlessly into patients’ lives and clinicians’ workflows in a way that relieves burden and addresses unmet needs [23]. Therefore, we have designed this pilot study to field-test the logistical aspects of Orchestra implementation in a real-life clinical setting in preparation for a larger, more definitive study to test the effectiveness of this type of technology-enabled coproduction in improving patient outcomes and health care value [9]. Our approach is focused not only on testing the feasibility and acceptability of the Orchestra technology, but also on refining the processes to optimize how Orchestra integrates into patients’ and families’ lives and clinicians’ workflows, and to support patients and clinicians in using this technology to collaboratively produce better health care outcomes.

This study has several limitations. While broad inclusion criteria encourage a wide range of participants, we are recruiting from patients seen in two clinics at a single hospital. This convenience sample may not be representative of other clinics or the larger populations of children with IBD and CF. In addition, while measures of acceptability and feasibility can be obtained by directly measuring interaction with the app, our core measures of impact are being obtained from surveys of patients/caregivers and clinicians following clinical encounters. We have maximized research processes to optimize survey completion rates. However, our results have the potential to be negatively impacted by low response rates. Due to the pilot nature of this study and the desire to obtain feedback across a narrow range of acceptability [24] and feasibility dimensions, we are not explicitly using the Unified Theory of Acceptance and Use of Technology (UTAUT) model. Future larger-scale studies and any planned implementation efforts of the Orchestra technology should examine the UTAUT model. Furthermore, there are multiple facets of feasibility and acceptability that are not being measured due to resource constraints and concerns about participant burden. For example, while we are tracking the frequency of technology bugs, we are not including robust measures of time and resources spent on technology support (eg, how much support was needed to address connectivity issues or data loss). Finally, this study is not designed to assess impact on clinical outcomes. Given the short duration of participant involvement (6-months), we do not anticipate seeing changes in longer-term health outcomes. Despite this limitation, if we observe improvements in short-term outcomes as measured in this study, we would feel more confident proceeding with a larger study designed to measure impact on clinical outcomes and health care value.

This study is novel in testing a technology coupled with processes that facilitate patient and clinician collaboration. This approach has the potential to shift the way mHealth technology is developed to support a model whereby equal attention is paid to the technology and the humans (patients, caregivers, and clinicians) who are the true agents of transformation.

## Appendix

Multimedia Appendix 1 Orchestra screen shots.

Multimedia Appendix 2 Examples of participant trackers.

Multimedia Appendix 3 Orchestra participant quick start guide.

## Abbreviations

BMI: body mass index
CCHMC: Cincinnati Children’s Hospital Medical Center
CF: cystic fibrosis
EHR: electronic health record
FEV1: Forced Expiratory Volume in 1 second
IBD: inflammatory bowel disease
ID: identification
IRB: Institutional Review Board
mHealth: mobile health
PHI: protected health information
PRO: patient-reported outcome
PVP: previsit plan
RC: research coordinator
REDCap: Research Electronic Data Capture
SMS: short messaging system
SPC: statistical process control
UTAUT: Unified Theory of Acceptance and Use of Technology
VPC: virtual private cloud
