# Supplementary material for: Protocol of a Pilot Study of Technology-Enabled Coproduction in Pediatric Chronic Illness Care
Source: JMIR Res Protoc. 2017 Apr 28;6(4):e71. doi: 10.2196/resprot.7074 (PMC5429432; doi:10.2196/resprot.7074)
Supplement: Multimedia Appendix 1 [file resprot_v6i4e71_app1.pdf]

Orchestra Mobile Application: Used by participants

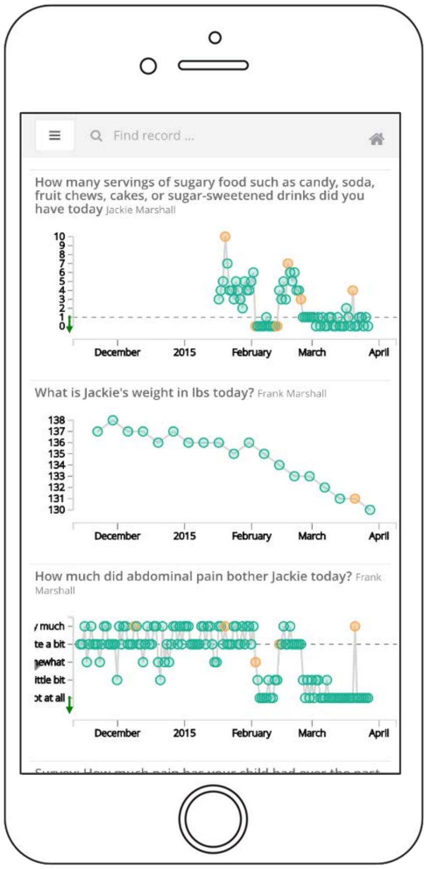

Real-time Tracker Data Visualization

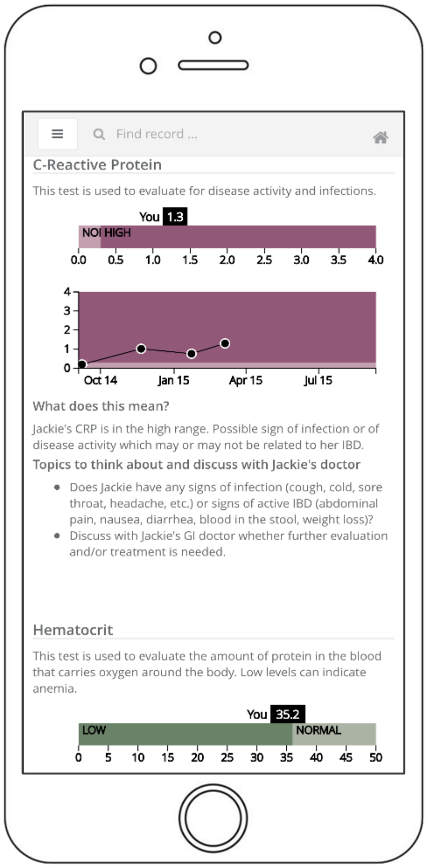

PVP: Health Metrics Feedback

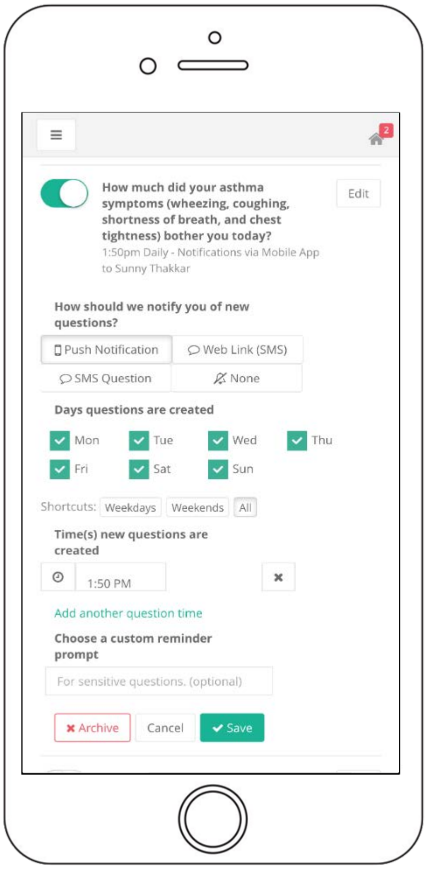

Tracker Editing Function

Orchestra Web Application: Used by clinicians

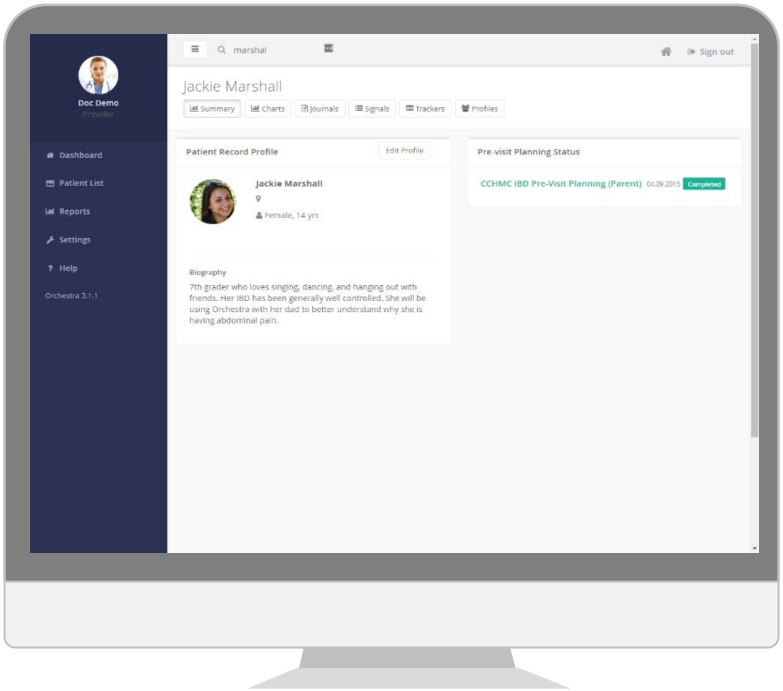

Patient Summary

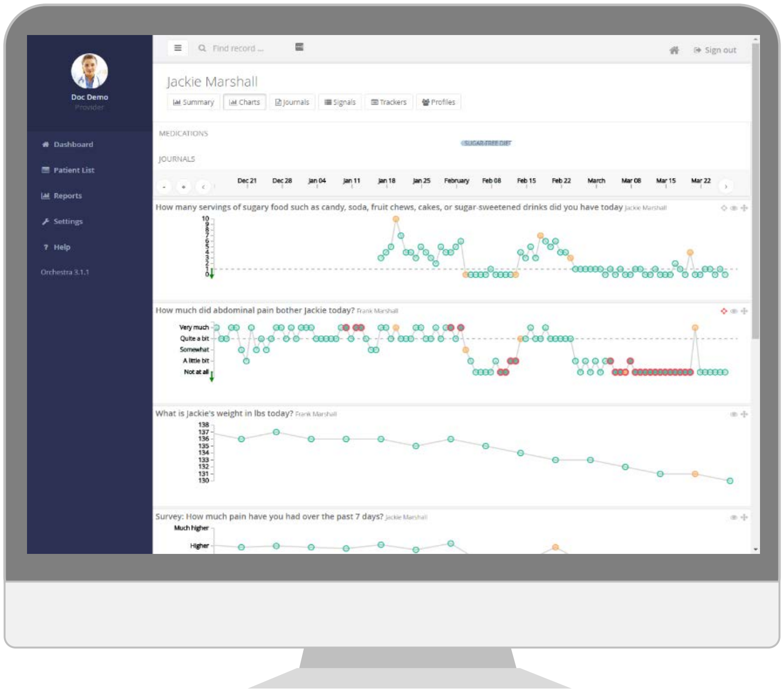

Patient Charts
