# Supplementary material for: Protocol of a Pilot Study of Technology-Enabled Coproduction in Pediatric Chronic Illness Care
Source: JMIR Res Protoc. 2017 Apr 28;6(4):e71. doi: 10.2196/resprot.7074 (PMC5429432; doi:10.2196/resprot.7074)
Supplement: Multimedia Appendix 2 [file resprot_v6i4e71_app2.pdf]

Orchestra Mobile Application: Examples of Participant Trackers

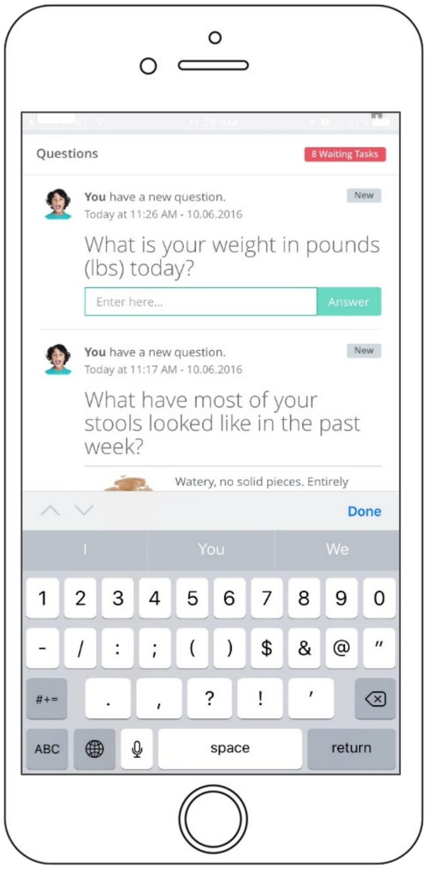

Integer/Decimal Data Entry

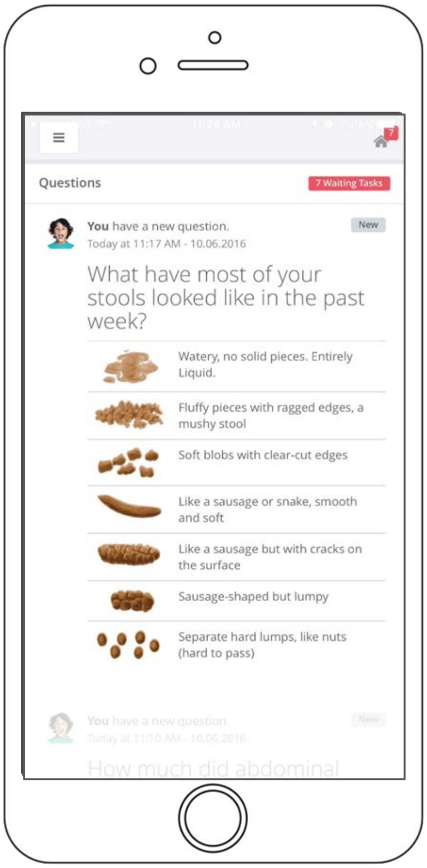

Choice Data Entry with Graphic

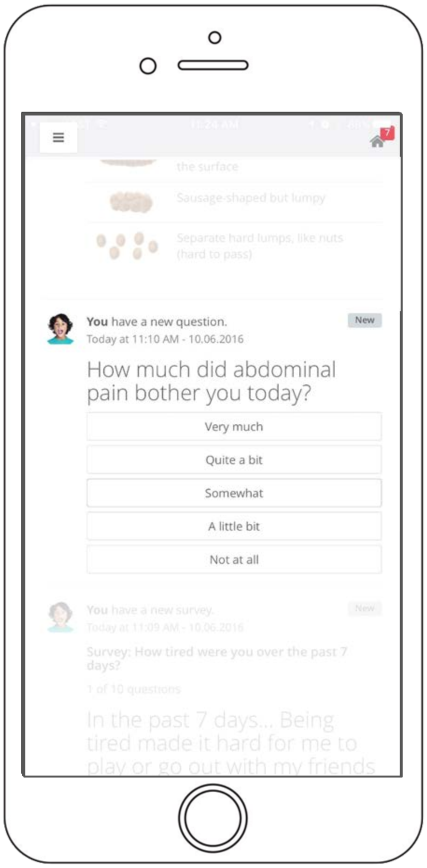

Choice Data Entry with Text
