# Supplementary material for: Protocol of a Pilot Study of Technology-Enabled Coproduction in Pediatric Chronic Illness Care
Source: JMIR Res Protoc. 2017 Apr 28;6(4):e71. doi: 10.2196/resprot.7074 (PMC5429432; doi:10.2196/resprot.7074)
Supplement: Multimedia Appendix 3 [file resprot_v6i4e71_app3.pdf]

ORCHESTRA

MOBILE APP QUICK START GUIDE

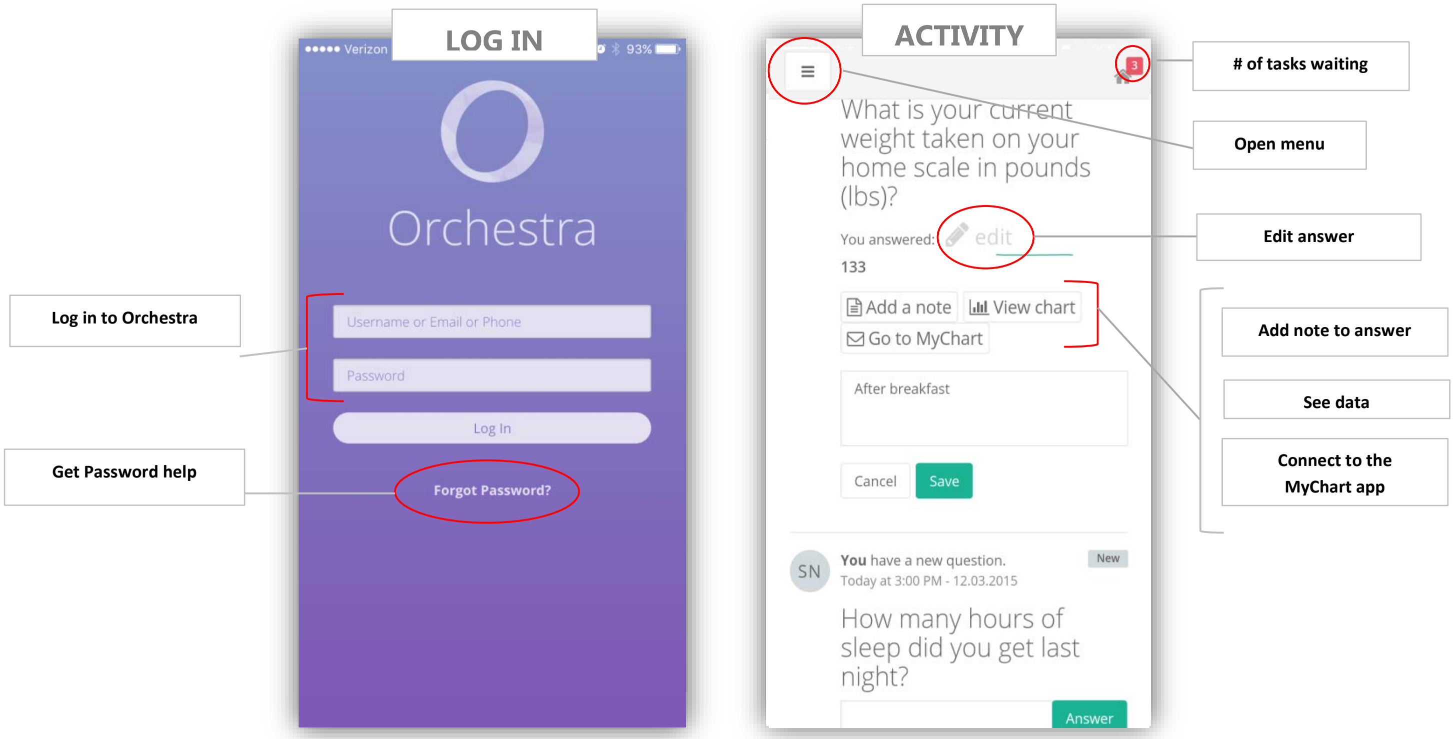

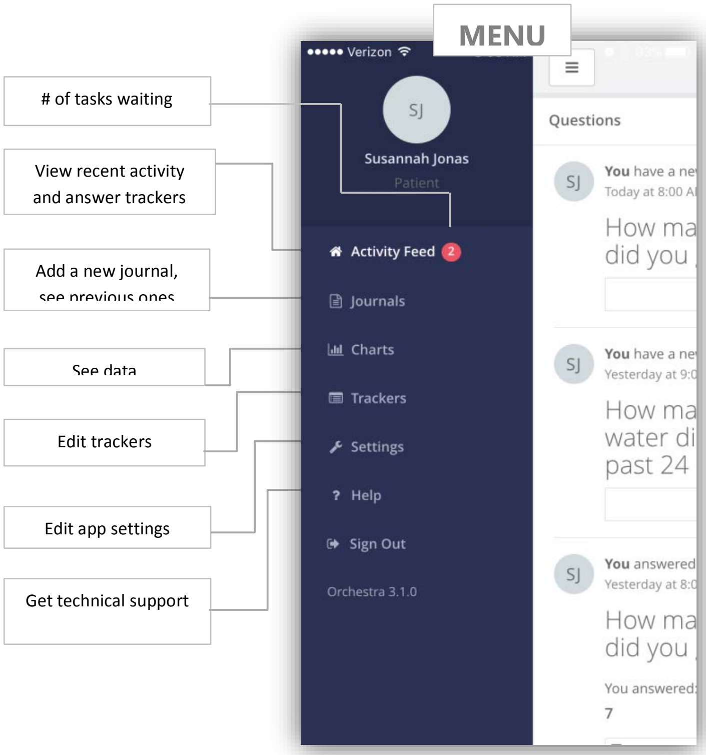

MENU
# of tasks waiting
View recent activity and answer trackers
Add a new journal, see previous ones
See data
Edit trackers
Edit app settings
Get technical support
Verizon
SJ
Susannah Jonas
Patient
Activity Feed 2
Journals
Charts
Trackers
Settings
Help
Sign Out
Orchestra 3.1.0
Questions

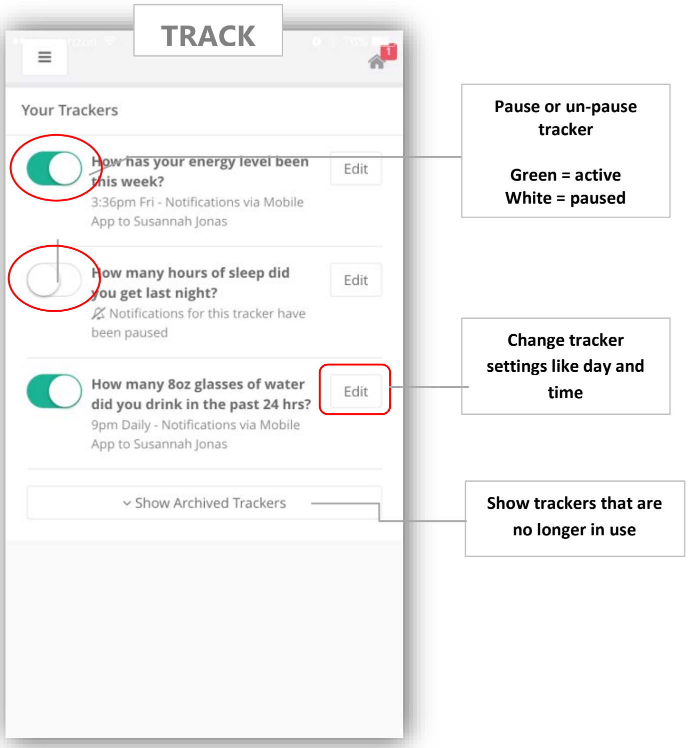

TRACK
Your Trackers
How has your energy level been this week?
3:36pm Fri - Notifications via Mobile App to Susannah Jonas
Edit
How many hours of sleep did you get last night?
Notifications for this tracker have been paused
Edit
How many 8oz glasses of water did you drink in the past 24 hrs?
9pm Daily - Notifications via Mobile App to Susannah Jonas
Edit
Show Archived Trackers
Pause or un-pause tracker
Green = active
White = paused
Change tracker settings like day and time
Show trackers that are no longer in use

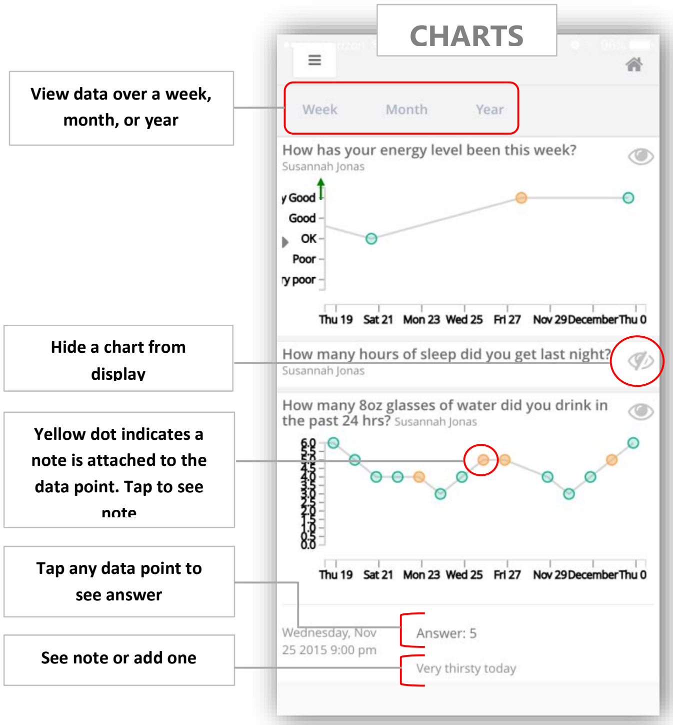

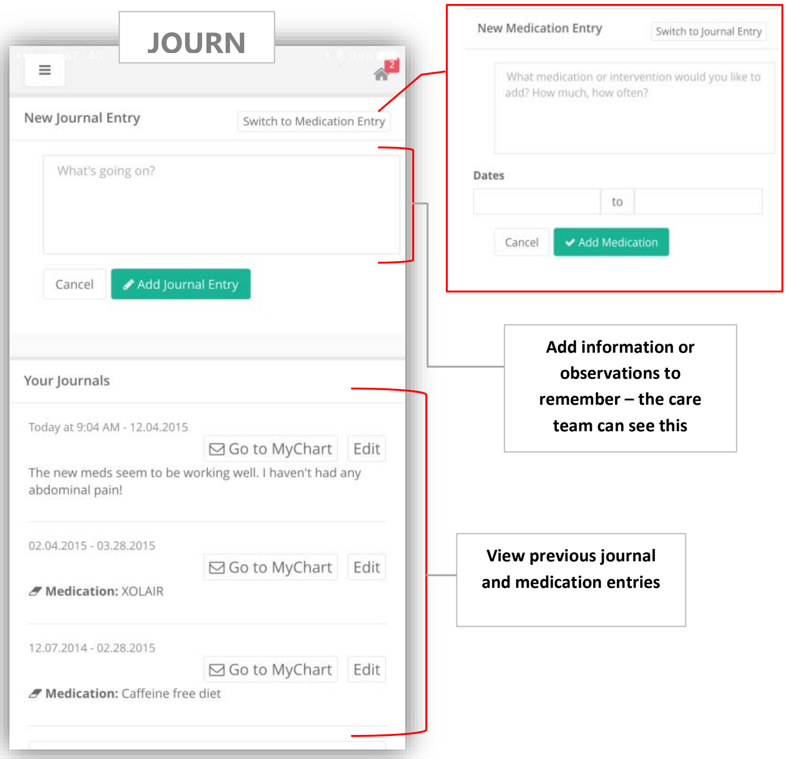

To view your Orchestra account in more detail from your laptop or desktop, please visit www.orchestra.md

Username: ______________________________

Password: ______________________________
